# Does conservation account for splicing patterns?

**DOI:** 10.1186/s12864-016-3121-4

**Published:** 2016-10-07

**Authors:** Michael Wainberg, Babak Alipanahi, Brendan Frey

**Affiliations:** 1 Department of Electrical and Computer Engineering, University of Toronto, 10 King’s College Road, Toronto, M5S 3G4 Canada; 2 Centre for Cellular and Biomolecular Research, University of Toronto, 160 College Street, Toronto, M5S 3E1 Canada; 3 Program on Genetic Networks and Program on Neural Computation & Adaptive Perception, Canadian Institute for Advanced Research, 180 Dundas Street West, Suite 1400, Toronto, M5G 1Z8 Canada

**Keywords:** Alternative splicing, Conservation, Splicing regulation

## Abstract

**Background:**

Alternative mRNA splicing is critical to proteomic diversity and tissue and species differentiation. Exclusion of cassette exons, also called exon skipping, is the most common type of alternative splicing in mammals.

**Results:**

We present a computational model that predicts absolute (though not tissue-differential) percent-spliced-in of cassette exons more accurately than previous models, despite not using any ‘hand-crafted’ biological features such as motif counts. We achieve nearly identical performance using only the conservation score (mammalian phastCons) of each splice junction normalized by average conservation over 100 bp of the corresponding flanking intron, demonstrating that conservation is an unexpectedly powerful indicator of alternative splicing patterns. Using this method, we provide evidence that intronic splicing regulation occurs predominantly within 100 bp of the alternative splice sites and that conserved elements in this region are, as expected, functioning as splicing regulators. We show that among conserved cassette exons, increased conservation of flanking introns is associated with reduced inclusion. We also propose a new definition of intronic splicing regulatory elements (ISREs) that is independent of conservation, and show that most ISREs do not match known binding sites or splicing factors despite being predictive of percent-spliced-in.

**Conclusions:**

These findings suggest that one mechanism for the evolutionary transition from constitutive to alternative splicing is the emergence of *cis*-acting splicing inhibitors. The association of our ISREs with differences in splicing suggests the existence of novel RNA-binding proteins and/or novel splicing roles for known RNA-binding proteins.

**Electronic supplementary material:**

The online version of this article (doi:10.1186/s12864-016-3121-4) contains supplementary material, which is available to authorized users.

## Background

Alternative splicing, the production of multiple mRNA isoforms from a single gene, is critical to the generation of biological complexity and the differentiation of both tissues and species [1]. Consequently, there has been great interest in recent years in developing *in silico* models of the *splicing code* – the interactions of *cis* and *trans* regulatory elements – from simpler biological features such as genetic sequence, nucleosome positions and RNA secondary structure [2, 3]. Ideally, a splicing model should be able to make several types of predictions: the ‘absolute’ *percent-spliced-in* *Ψ* of any exon in various tissues, *Δ* *Ψ* between tissues, the impact of mutations on *Ψ* [4], and binding sites for RNA-binding proteins (RBPs) that affect splicing [5]. Notably, none of these goals requires the model to actually mimic the inner workings of the cell, and most metrics used to evaluate the quality of a model’s predictions do not take into account its biophysical fidelity.

It has long been known that alternative splicing is associated with modified evolutionary conservation of both exons [6] and their flanking introns [7]. Modrek and Lee [6] found that newly created exons (those with non-conserved splice junctions) have low *Ψ* and hypothesized that this served a useful evolutionary purpose, by allowing the exon to accumulate beneficial mutations without the organism losing the benefits of the original protein in the meantime. Sorek and Ast [7] noted that alternatively spliced exons are disproportionately likely to have conserved flanking introns, and identified that one abundant k-mer in conserved downstream introns had known *cis*-regulatory properties.

The role of intronic conservation extends to tissue-specific splicing regulation as well. Sugnet et al. [8] found that exons with high *Δ* *Ψ* between brain or muscle and other tissues tended to have highly conserved flanking introns. Yeo et al. [9] discovered that conserved Fox and Nova motifs in introns are associated with higher *Ψ* in brain tissue. Wang et al. [10] found that exons with ‘switch-like’ *Δ* *Ψ*> 0.5 between any pair of tissues have increased conservation in flanking introns.

Computational models of splicing often depend on conservation for their accuracy. A previous study on alternative splicing modelling [2] found that a metric of model quality increased by one-third when conservation was incorporated into the model. The most accurate existing models of alternative splicing [4, 11, 12] also rely heavily on conservation. These models train neural networks on over 1000 ‘hand-crafted’ features, including motif counts, position weight matrix (PWM) correspondences, sequence lengths, RNA secondary structure, nucleosome positions, and translatability and frameshift features. In these models, conservation is used both in raw form, as averages over the first 100 bp of each flanking intron (*average conservation*), and to weight motif counts. The underlying assumption is that conservation is mostly useful to indicate the overall level of *cis* elements in flanking introns (average conservation) and to determine which occurrences of interesting motifs are actually relevant for splicing (conservation-weighted motif counts).

This article introduces several computational models of splicing that depend on conservation, with the goal of understanding the evolution of alternative splicing. Some previous studies of alternative splicing and conservation [13–15] analyze the conservation of alternative splicing patterns between species. Instead, we prefer to focus on the conservation of the sequence near alternative splice sites, as this incorporates flanking introns into the analysis and provides more fine-grained insights into the differing roles of conservation in various regions of the sequence.

## Results and discussion

**Fig. 1:**
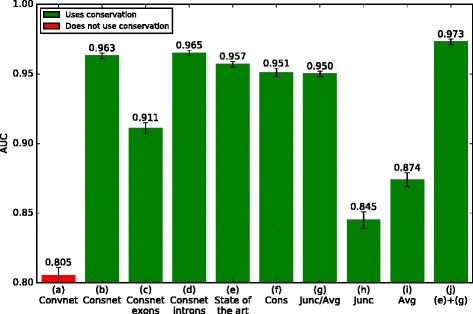
AUC of various alternative splicing models. (a) A convolutional DNN trained on sequences up to 384 bp from each of the four splice sites involved in cassette splicing (8 × 384 bp). (b) Same as (*a*), weighting the post-convolutional feature map by conservation (8 × 384 bp). (c) Same as (*b*), using only the 100 bp at each end of the cassette exon (2 × 100 bp). (d) Same as (*c*), using only the first 100 bp of each flanking intron (2 × 100 bp). (e) State of the art: the method of [11] (1393 features). (f) A DNN trained on only the conservation of the regions in (*d*) (2 × 100 features). (g) A DNN trained on junction conservation divided by average conservation over 100 bp (2 features). (h) A DNN trained on junction conservation (2 features). (i) A DNN trained on average conservation over 100 bp (2 features). (j) A DNN trained on the combined features of (*e*) and (*g*) (1393 + 2 features)

We constructed several computational models of alternative splicing in humans from RNA-Seq data and compared the accuracy of their absolute *Ψ* predictions, measured as the ability to distinguish cassette exons with high *Ψ* (> 2/3) from those with low *Ψ* (< 1/3) for all tissues simultaneously (Fig. 1). Strikingly, one technique which surpasses all previous techniques (Fig. 1(b)) does not use any hand-crafted features at all: instead, it learns directly from the sequence and conservation track (33-way mammalian phastCons). No additional information is provided besides the locations of the splice sites.

However, even this minimalist model, which we call ConsNet, or conservation-weighted convolutional neural network, is substantially more complex than what is necessary to predict *Ψ*. Nearly equivalent performance was obtained with a very simple neural network model trained on just two features for each exon: the conservation on the intronic side of each of its splice junctions (*junction conservation*) normalized by the average conservation over the first 100 bp of the corresponding flanking introns (*average conservation*), hereinafter called the *Junc/Avg* model (Fig. 1(g)). (The Junc/Avg model is almost equivalent to training ConsNet on only the first base into the intron, since ConsNet’s conservation track was also normalized by average conservation).

When trained in a simple logistic regression with all of the hand-crafted features from previous models, these two features have the highest weights (Table 1), emphasizing that the Junc/Avg features are individually more useful than any previous features. Further, adding the Junc/Avg features to the previous features improves AUC by 1.6 % (compare Fig. 1(e) and (j)), indicating that Junc/Avg provides substantial new information not captured by any previous features. In particular, Junc/Avg is not merely a proxy for splice site strength: a deep learning model trained to predict *Ψ* from the MaxEntScan [16] splice site scores of the two alternative splice sites and the two neighbouring constitutive splice sites achieves an AUC of only 0.643 ± 0.007, subtantially lower than the Junc/Avg model.

**Table 1:** Junc/Avg features are more predictive than any previous features

Feature	Weight
**Downstream Junc/Avg**	**0.904**
**Upstream Junc/Avg**	**0.747**
C1AC2 Translatability	0.691
C1A Translatability	0.567
C1C2 Translatability	-0.473
Log length of A	0.424
Upstream splice site strength	0.345

Weights of the top 7 features in a logistic regression of 1393 features based on the 1014 features used in [2] and the Junc/Avg features. The Junc/Avg features (shown in bold) are individually more predictive of *Ψ* than any of the features used previously

While the Junc/Avg model is worse than [11] at predicting the absolute *Ψ* values of events with intermediate *Ψ* (Table 2), it still performs respectably well even at this task.

**Table 2:** The state of the art outperforms the Junc/Avg model at intermediate *Ψ* prediction

*Ψ*	Events	Junc/Avg	[11]
All	6871	0.643 (*p*< 1e-308)	0.647 (*p*< 1e-308)
0.1-0.9	319	0.261 (*p*< 2e-06)	0.293 (*p*< 1e-07)
0.2-0.8	159	0.117 (*p*< 0.1)	0.200 (*p*< 0.01)
0.3-0.7	83	-0.134 (*p*< 0.2)	-0.174 (*p*< 0.1)
0.4-0.6	9	-0.778 (*p*< 0.01)	0.050 (*p*< 0.9)

Performance (Spearman correlation with *Ψ*) of the Junc/Avg model and [11] at predicting the absolute *Ψ* values of intermediate- *Ψ* events. An event is defined to fall into a particular *Ψ* range if both *Ψ*+*σ* and *Ψ*−*σ* are inside the range (where *σ* is the standard deviation of *Ψ* across tissues), i.e. if it is at least one full standard deviation inside

The Junc/Avg model appears to be an exceptionally concise summary of phastCons conservation information: it performs as well as a neural network trained on the full 100 bp of intronic conservation (compare Fig. 1(f) and (g)); and it is the simplest model to do so, as junction and average conservation individually perform far worse (Fig. 1(h)/(i)).

The utility of junction and average conservation is not limited to phastCons. GERP++ [17], a measure of purifying selection derived from multiple sequence alignments, is not normalized to be between 0 and 1 so the heuristic of dividing junction by average conservation is not applicable. However, a model trained to predict *Ψ* from the upstream and downstream GERP++ junction and average scores individually (4 features) obtains an AUC of 0.948 ± 0.003, comparable to using phastCons.

Nonetheless, there are limits to the power of conservation. The Junc/Avg model performs worse than existing models at predicting *Δ* *Ψ* between tissues: the Spearman correlation between its predictions and the true tissue differences, concatenated over all tissue pairs and excluding pairs of measurements that are statistically indistinguishable (*σ*(*Δ* *Ψ*)>|*Δ* *Ψ*|), is a mere 0.017, compared to 0.072 using the method described in [11]. Even so, the Junc/Avg model does well at predicting *Ψ* for exons with substantial tissue differences in inclusion (max cross-tissue |*Δ* *Ψ*|>10 *%*), with an AUC of 0.883 ± 0.007.

The Junc/Avg model is also too simple to predict differences in *Ψ* due to mutations, not least because it does not have access to the sequence and thus has no conception of what a mutation is. Even for absolute *Ψ* prediction, the sequence contains information beyond what is provided by conservation: giving the model access to the sequence improves AUC (area under the receiver-operating characteristic) by 1.4 % (compare Fig. 1(d) and (f)).

On a more fundamental level, this model does not capture as much biophysical information as previous models, which can predict the effect of *trans* elements on splicing. Xiong et al. [4] found that removing Muscleblind-like RBPs from their model had a similar effect on *Ψ* to knocking down these RBPs in actual cells.

### Intronic splicing regulation occurs predominantly within 100 bp of the alternative splice sites

Comparing Fig. 1(c) and (d), we see that ConsNet performs worse at predicting absolute *Ψ* from the 100 bp at each end of the cassette exon than from the first 100 bp of each flanking intron, perhaps because exonic conservation is not a pure indicator of splicing regulation as it is confounded by effects on protein function. With this in mind, we focus on intronic splicing regulation rather than exonic regulation for the remainder of our analysis.

**Fig. 2:**
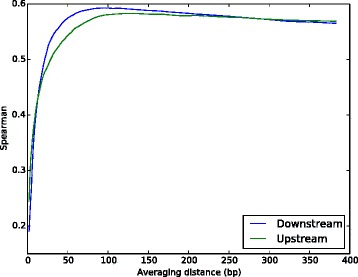
Most intronic splicing regulation occurs within 100 bp of the splice site. Correlation between junction/average conservation and tissue-averaged *Ψ* as the averaging window is increased from 1 to 384 bp of the flanking introns nearest the splice site. The correlation peaks at 132 bp for the upstream splice site and 92 bp for the downstream splice site

Is there anything special about 100 bp? Figure 2 shows the correlation between junction/average conservation and *Ψ* as the averaging window is increased from 1 to 384 bp into the flanking intron. For upstream introns, the maximum correlation occurs at an averaging window of 132 bp; for downstream introns, 92 bp. Beyond these distances, incorporating additional distal conservation information into the average only degrades the prediction.

To confirm that using only the first 100 bp of each flanking intron does not miss much information, note that once ConsNet is provided with these 200 bp, it does not help the prediction any further to provide it with the conservation and sequence of an entire 384 bp on *both* the intronic and exonic sides of the splice sites *and* the same 768-bp region around the two nearest constitutive splice sites (compare Fig. 1(b) and (d)).

Under the assumption that conserved sequences near introns are predominantly splicing regulators, these results provide evidence that most intronic splicing regulation occurs within about 100 bp of the splice site in both upstream and downstream introns. This does not negate the fact that some important intronic splicing regulation occurs further away from the splice site [18]; however, such distal regulation appears to be the exception rather than the rule.

### New exons, and old exons with conserved *cis* elements, have reduced *Ψ*

**Fig. 3:**
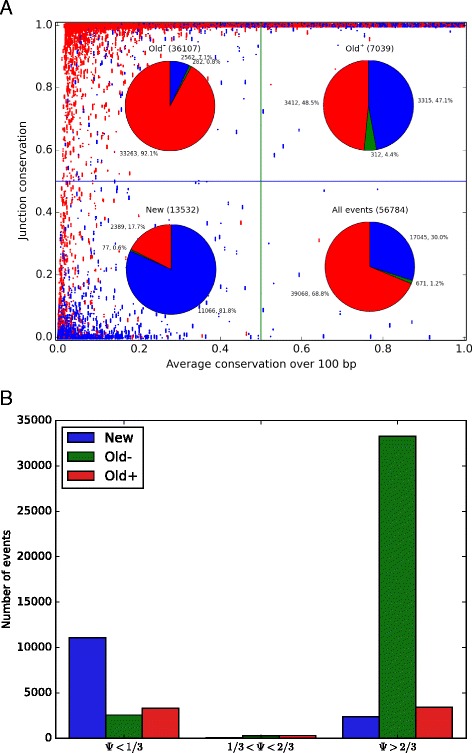
Junction versus average conservation. **a**) *Upstream* junction versus average conservation for all high-confidence (*σ*(*Ψ*)< 0.1) *high* *Ψ* (*red*) and *low* *Ψ* (*blue*) events (*downstream* results are similar). 99.8 % of all events fall into one of 3 regimes: high (> 0.5) junction and *low* average conservation (Old^-^), high junction and average conservation (Old^+^), and low junction and average conservation (New). Small Gaussian noise was applied in the y direction to avoid superimposing all tissues for each exon. Pie charts of *Ψ* for each regime and the whole dataset, also including medium *Ψ* events (*green*), are superimposed. **b**) The same data broken down first by *Ψ* range and then by conservation regime

We next investigated why Junc/Avg conservation is significantly more informative than either Junc or Avg on their own. Fig. 3 shows a scatter plot of upstream junction versus average conservation for high *Ψ* (red) and low *Ψ* (blue) events (as for most of the following analysis, downstream results are similar). The events are divided into 3 regimes based on whether they have high (> 0.5) or low junction or average conservation:

1. Old^–^: old exons with few conserved *cis* elements (high junction, low average)
2. Old^+^: old exons with many conserved *cis* elements (high junction, high average)
3. New: new exons (low junction, low average)

The size of the fourth category, with low junction and high average conservation, is negligible with only 0.2 % of events. We note that old and new are relative terms and are on the time scale of the most recent common ancestor of the species included in the conservation track.

As seen from the pie charts of Fig. 3, events in Old^-^ overwhelmingly have high *Ψ*, those in New mostly have low *Ψ*, and those in Old^+^ are evenly split between high and low *Ψ* with most exons exhibiting differential regulation between cell types (see Additional file 1: Section 1). This explains why Junc/Avg is so effective: junction conservation distinguishes low *Ψ* New from high *Ψ* Old^-^, average conservation distinguishes high *Ψ* Old^-^ from lower *Ψ* Old^+^, but Junc/Avg does both because it assigns similarly low values to both New and Old^+^, which both have relatively low *Ψ* (note that all events along the line *y*=*x* have the same Junc/Avg value).

The success of both ConsNet and the Junc/Avg model, compared to previous models of alternative splicing, is primarily attributable to the use of junction conservation. Previous models assumed that only the conservation of intronic *cis* elements was important, but our results show that conservation of the splice junction, which is related to the evolutionary age of the exon, is also extremely predictive of splicing.

### Conserved *cis* elements are associated with reduced *Ψ* across multiple evolutionary timescales

The inverse correlation between conserved *cis* elements and *Ψ* is not limited to conservation across mammalian species. Within each of the regimes New, Old^–^ and Old^+^ – which, recall, refer to mammalian conservation – we compared the *Ψ* values of exons with high versus low average primate conservation over 100 bp of flanking introns (Table 3). Among Old^–^ exons, exons with high average primate conservation – which recently developed conserved *cis* elements – have lower *Ψ* than exons which never developed many conserved *cis* elements. Conversely, among Old^+^ exons, exons with low average primate conservation – which recently *lost* conserved *cis* elements – have higher *Ψ* than exons that retained them.

**Table 3:** Intronic *cis* elements that diverged farther in the past have greater differences in splicing patterns

Mammalian conservation	Average primate conservation	Mean *Ψ*
Old^-^	Low	0.728 ± 0.003
	High	0.63 ± 0.01
Old^+^	Low	0.56 ± 0.02
	High	0.500 ± 0.007
New	Low	0.390 ± 0.004
	High	0.37 ± 0.03

Mean and standard error of *Ψ* for events in each regime, broken down further by whether the average primate conservation over 100 bp of the upstream flanking intron is high (> 0.5) or low (downstream results are similar). Events in Old^-^ have higher *Ψ* than those in Old^+^; within each of these regimes, events with low average primate conservation also have higher *Ψ*

This indicates that an increase in the conservation of *cis* elements over both the evolutionary timescale of primates (∼ 50 million years) and mammals (∼ 200 million years) is associated with a reduction in *Ψ*. However, these two timescales are not equally important: exons with many conserved *cis* elements in mammals but not in primates (Old^+^ Low) still have lower *Ψ* than those with many conserved *cis* elements in primates but not in mammals (Old^-^ High). This indicates that mammalian conservation of *cis* elements has a more pronounced relationship with *Ψ* than primate conservation, and that exons with intronic *cis* elements that diverged farther in the past have greater differences in splicing patterns.

### Conserved flanking introns are associated with increased tissue-specific splicing, particularly in the brain

Echoing the relationship demonstrated by [10] between intronic conservation and tissue-specific splicing (see Background), Table 4 shows that the proportions of low, medium and high *Ψ* exons have much more inter-tissue variance in Old^+^ than in Old^–^. This suggests that conserved flanking introns cause tissue differences in splicing patterns. There is a particularly large difference in splicing between brain tissue and other tissues in Old^+^ that barely appears in the other regimes, indicating that the cassette exons responsible for giving the brain its unique phenotypic qualities lie disproportionately in Old^+^.

**Table 4:** Conserved flanking introns are associated with greater tissue differences in splicing patterns

Tissue	Old^-^	Old^+^	New
Adipose	8/0/91	51/4/44	82/0/17
Adrenal	6/0/92	48/4/47	78/0/20
Brain	6/1/91	**35 / 8 / 55**	86/0/13
Breast	7/0/92	46/3/50	83/0/16
Colon	7/0/91	50/3/45	83/0/15
Heart	6/0/92	43/5/50	85/0/13
Kidney	6/0/92	50/2/46	81/0/17
Liver	6/0/93	49/2/48	80/0/19
Lung	7/0/91	52/2/44	80/0/18
Lymph	7/0/92	55/2/42	81/0/17
Ovary	8/0/90	48/5/46	82/0/17
Prostate	6/0/92	49/2/47	81/0/17
Skel. muscle	5/0/94	36/3/59	81/1/16
Testes	6/1/91	45/5/49	81/0/17
Thyroid	7/1/91	44/6/48	80/0/18
White blood	6/0/92	48/2/48	75/0/23
**Std. dev.**	**0.8/0.3/0.8**	**2.3/0.3/2.4**	**4.2/1.9/5.1**

Percentages of low/medium/high *Ψ* values among exons in each regime and tissue, and standard deviations of each percentage across tissues, for upstream flanking introns (downstream results are similar). Note the particularly large difference between brain and other tissues in Old^+^ (bolded)

However, tissue-specific regulation is not the primary reason why Old^+^ is evenly split between high and low *Ψ*. Among exons in Old^+^ with high-confidence measurements in at least half of tissues, 74 % upstream and 73 % downstream are either high- *Ψ* or low- *Ψ* in *every* tissue. In other words, only about a quarter of Old^+^ exons display any substantial tissue-specific regulation.

### A new definition of intronic splicing regulatory elements

We next studied the relationship of k-mer counts to intronic conservation and splicing. We restricted our analysis to between 15 and 100 bp into the intron from the splice site (the *15-100 bp region*), since regions beyond 100 bp are less important for splicing regulation, and using the first 15 bp adjacent to the splice site would confound our analysis with splicing consensus sequences.

Yeo et al. [9] defined *intronic splicing regulatory elements* (ISREs) as k-mers that are significantly more conserved than the background in the nearest 400 bp to the splice site of both upstream and downstream flanking introns. Voelker and Berglund [19] also used conserved regions as a basis for finding novel ISREs. However, conservation alone is not enough to predict whether a k-mer is associated with increased or decreased splicing, i.e. whether it is an *intronic splicing enhancer* (ISE) or an *intronic splicing silencer* (ISS).

An intuitive definition of an ISE (or ISS) is a k-mer with the property that events containing the k-mer have a higher (or lower) *Ψ* than events that do not. However, this definition does not account for differences in mono- and dinucleotide frequencies between high- and low- *Ψ* events, and would for instance result in GC-rich k-mers being labelled as ISEs and AT-rich k-mers as ISSs because GC-rich introns are associated with increased splicing. To control for this, we trained a linear regression model to predict tissue-averaged *Ψ* from mono- and di-nucleotide frequencies and considered the residuals of this model – the difference between the true and predicted *Ψ* values for each event, which are assumed to be the component of *Ψ* not attributable to mono- and di-nucleotide frequencies – which we call *residual* *Ψ*. We define a k-mer as an ISE if residual *Ψ* is significantly higher in events containing the k-mer than in events not containing the k-mer at FDR [20] *q*< 0.05, and as an ISS if residual *Ψ* is significantly lower, according to a Mann-Whitney test. Under this definition, there are 7 upstream/16 downstream ISE 6-mers and 35 upstream/85 downstream ISS 6-mers, or 143 ISRE-region pairs in total (see Additional file 1: Section 2 and Additional file 2 for a list).

To validate this definition, we trained a neural network to predict absolute *Ψ* using only ISRE counts in the upstream and downstream 15–100 bp regions, and achieved an AUC of 0.644 ± 0.006, increasing to 0.810 ± 0.006 when two additional features were included to account for nonsense-mediated decay (NMD), Translatable.C1C2 and Translatable.C1AC2 (see [12]). (For this experiment alone, we defined ISREs based on the correlation across only exons not in the test set to avoid bias, leading to a slightly smaller list of 132 ISRE-region pairs.) [9]’s 296 upstream and 278 downstream 6-mers were less useful at predicting *Ψ*, performing no better than the same number of random 6-mers (Table 5), despite containing over 4 times as many 6-mers as our list.

**Table 5:** Our ISREs predict *Ψ* more accurately than [9]’s

	Our ISREs *n* = 132	[9] *n* = 574	Random *n* = 574
Counts	0.644 ± 0.006	0.601 ± 0.008	0.609 ± 0.008
+NMD	0.810 ± 0.006	0.788 ± 0.005	0.805 ± 0.006
Cons	0.693 ± 0.006	0.608 ± 0.008	0.615 ± 0.007

AUCs for absolute *Ψ* prediction using k-mer counts, counts plus two nonsense-mediated decay (NMD) features from [12], and conservation-weighted counts for various k-mer sets. The conservation weight of each k-mer instance is the minimum conservation over its k bases, to avoid counting non-functional k-mer instances that partially overlap functional ones. Our ISREs predict *Ψ* more accurately than [9]’s, which perform no better than the same number of random k-mers

Interestingly, there is little overlap between our and [9]’s ISREs: upstream, only 2 of our ISREs (CCTCAG and TGAGTA) overlap [9]’s (compared to an expected value of 296 * 42/4096 = 3.0 if the two sets were selected randomly), 7.2 % of our ISREs’ instances and 5.1 % of our ISREs’ total basewise conservation; downstream, only 4 of our ISREs (TCTGAA, TTAAGA, GTATTT and ATTAGA) overlap (compared to an expected value of 278 * 101/4096 = 6.9 if random), 7.1 % of their instances and 7.2 % of their conservation overlap. One hypothesis for the lack of overlap is that the two definitions may capture different types of ISREs: perhaps our ISREs are moderately functional in a wide variety of locations, while [9]’s are highly functional but only in conserved locations, i.e. in specific genetic contexts. However, using conservation-weighted k-mer counts instead of raw counts, [9]’s ISREs are still less predictive than ours (Table 5), which seems to contradict this hypothesis.

There is also little overlap between our ISREs and known RBP binding sites. We catalogued 793 6-mers which contain the NOVA binding site YCAY [21] or are subsequences of 7-mers found by [22] to have a high affinity (*E* score > 0.45) for at least one of 207 RBPs. We found that 12 of the 42 upstream ISREs and 14 of the 101 downstream ISREs matched one of these 793 6-mers, compared to the 42 * 793/4096 = 8.1 upstream and 101 * 793/4096 = 19.6 downstream ISREs that would be expected to match if the two sets were selected randomly. Additional file 1: Section 3 and Additional file 3 list the RBPs which had high affinity for each of the matching 6-mers.

### Common k-mers are more conserved in flanking introns

For each k-mer and region (upstream and downstream), we define three properties. First, a k-mer’s intronic *conservation enrichment* is the average conservation of all bases in the 15–100 bp region that are part of an instance of the k-mer, divided by the average conservation of the 15–100 bp region across all events. Conservation enrichment can be defined per event or globally across all events. If a k-mer has a conservation enrichment greater than 1, it is more conserved than a typical k-mer within 15–100 bp of the splice site.

Second, a k-mer’s *enrichment bias* is the Spearman correlation of its per-event conservation enrichment with residual *Ψ* across all events where the k-mer appears at least once. Positive values indicate conservation enrichment near high- *Ψ* exons and negative values indicate enrichment near low- *Ψ* exons.

Third, a k-mer’s *ISE/ISS character* is the Spearman correlation of its count in the 15–100 bp region with residual *Ψ* across events (to be consistent with the definition of conservation enrichment, we include only events where the k-mer appears at least once). k-mers with more positive (negative) Spearman correlations with residual *Ψ* are more likely to be ISEs (ISSs).

**Fig. 4:**
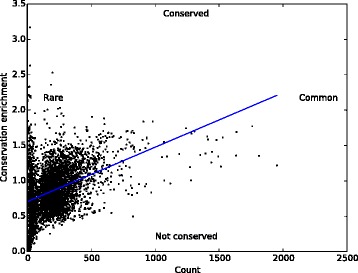
6-mer count versus conservation enrichment. Total count of each 6-mer versus conservation enrichment in *upstream* flanking introns (*downstream* results are similar). The high variation among k-mer counts (*σ* = 182) indicates substantial selection pressure on flanking introns. More common 6-mers tend to be more conserved (Spearman correlation 0.411, *p*< 1e-166). The most severely under-conserved k-mers (conservation enrichment less than one-third the background) are also extremely rare, appearing in a tight band along the *lower left* edge of the plot

Figure 4 shows the total count across events versus global conservation enrichment of each 6-mer in the upstream 15–100 bp region. The large variation among 6-mer counts (*σ* = 182) indicates substantial selection pressure on flanking introns, confirming what we already know from conservation: if introns were not under selection pressure, all counts would be approximately identical, aside from differences due to mono- and di-nucleotide frequencies. Common 6-mers are more conserved than rare ones (Spearman correlation 0.411, *p*< 1e–166), suggesting that some k-mers are inherently more useful than others and that variants disrupting the k-mers are under negative selection as a result. Conversely, the most severely under-conserved k-mers (conservation enrichment less than one-third the background) are also extremely rare, appearing in a tight band along the lower left edge of the plot.

### ISSs are more conserved near low *Ψ* exons; ISEs are more conserved near high *Ψ* exons

**Fig. 5:**
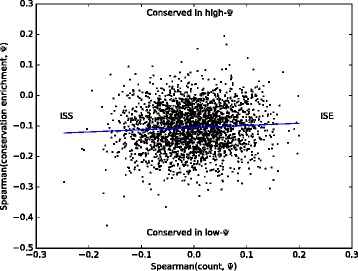
A ‘meta-correlation’ plot for 6-mers. The *x* coordinate of each 6-mer is the correlation across events where the k-mer appears of its count in the *upstream* 15–100 bp region (*downstream* results are similar) with tissue-averaged *Ψ* (ISE/ISS character), and the *y* coordinate gives the correlation of its conservation enrichment with *Ψ* (conservation bias). These two properties have a Spearman correlation of 0.0588 (*p*< 0.003) across all 6-mers. 6-mers appearing in fewer than 100 events or never appearing more than once in any event are not shown

Figure 5 compares the upstream ISE/ISS character and enrichment bias of each 6-mer across all events where the 6-mer appears at least once (1488 6-mers, or 36 % of all 6-mers, appeared in fewer than 100 events or never appeared more than once in any event and were excluded). Upstream, these two properties have a Spearman correlation of 0.059 (*p*< 0.003) across 6-mers; downstream, the correlation is 0.054 (*p*< 0.005). Hence, ISEs are more conserved near high *Ψ* exons and ISSs near low *Ψ* exons.

This further supports the view that k-mers have some degree of ‘inherent’ regulatory activity, in addition to activity that depends on location and context. Similar k-mers tend to become conserved in the introns flanking exons with similar *Ψ*, even though the k-mer occurs in different genetic contexts in each intron. If it is evolutionarily advantageous for an exon to have high *Ψ*, then ISEs will be under selection and eventually become conserved, and vice versa.

### *Ψ* appears to explain differences in ISS and ISE conservation patterns

In the analysis of the previous section, one might wonder whether *Ψ* was just an extraneous variable: perhaps ISE/ISS character and conservation bias are only correlated because k-mer count and conservation enrichment are themselves correlated. To rule out this possibility, we generated 1000 random permutations of *Ψ* values for each exon, then recalculated the two properties and their Spearman correlation. Upstream, the correlation was only larger in magnitude than 0.059 for 144 of these 1000 trials (*p*< 0.144); downstream, the correlation was larger than 0.054 for 175 of 1000 trials (*p*< 0.175). Though sub-significant, these results suggest that *Ψ* explains differences in ISS and ISE conservation patterns and that conserved intronic elements near splice sites are conserved because they regulate splicing.

### ISSs are more conserved than ISEs in new exons

We then compared the overall conservation of ISEs versus ISSs for various regimes, using a Mann-Whitney test to compare the distribution of conservation scores for every base in every ISE with the comparable distribution for ISSs (Table 6). Overall, upstream ISEs are more conserved than ISSs and downstream ISSs are more conserved than ISEs. Despite this difference, ISSs are more conserved near new exons both upstream and downstream, suggesting that intronic regulatory elements are at least partially responsible for the low percent inclusion of this set of exons.

**Table 6:** ISS vs ISE conservation across various exon sets

Regime	ISS med.	ISE med.	Diff	More cons‘d	p
(a) Upstream (35 ISSs, 7 ISEs)					
All	0.004	0.005	-0.001	ISEs	2e-39
Old^-^	0.001	0.003	-0.002	ISEs	4e-83
Old^+^	0.987	0.995	-0.008	ISEs	2e-5
New	0.007	0.002	0.005	ISSs	1e-56
High *Ψ*	0.001	0.004	-0.003	ISEs	1e-108
Low *Ψ*	0.013	0.011	0.002	ISEs	3e-5
(b) Downstream (85 ISSs, 16 ISEs)					
All	0.003	0.002	0.001	ISSs	4e-48
Old^-^	0.001	0.001	0	ISEs	1e-5
Old^+^	0.994	0.990	0.004	ISSs	0.0003
New	0.006	0.002	0.004	ISSs	4e-91
High *Ψ*	0.001	0.001	0	ISEs	6e-16
Low *Ψ*	0.007	0.006	0.001	–	0.4

Median of conservation scores across all bases that are part of any ISS/ISE, and which distribution of scores is larger according to a Mann-Whitney test. Upstream ISEs are more conserved than ISSs and downstream ISSs are more conserved than ISEs. Despite this difference, ISSs are more conserved near new exons both upstream and downstream, suggesting that intronic regulatory elements are at least partially responsible for the low percent inclusion of this set of exons

## Conclusions

There are several pathways by which alternative splicing can evolve [23]: exonization from non-coding regions, transition from constutitive splicing, and exon shuffling. Lev-Maor et al. [24] argued that the evolutionary transition from constitutive to alternative splicing is triggered by a weakening of the 5’ splicing consensus sequence. Our results suggest an additional mechanism for this transition: the emergence of conserved ISSs. Originally, old constitutive exons would be situated in Old^-^, with high *Ψ* and few conserved *cis* elements; if at some time it became beneficial for the exon to be alternatively spliced or have tissue-specific splicing patterns, conserved ISSs would eventually emerge and the exon would migrate to Old^+^.

We have identified 6-mer ISREs that are associated with *Ψ* in the upstream and downstream introns flanking alternatively spliced exons, listed in the Additional files. The relatively high predictive power of these ISREs suggests the existence of novel RNA-binding proteins and/or novel splicing roles for known RNA-binding proteins. Alternatively, some ISREs could influence splicing via RNA secondary structure or effects on transcription rate. These ISREs are promising candidates for future experimental study.

## Methods

The dataset used in this research consists of *Ψ* values across 16 tissues for 10689 cassette exons derived from RNA-Seq data and mapped to the hg19/GRCh37 human genome [25]. Low-confidence measurements (standard deviation of *Ψ*> 0.1) were pruned from the dataset when training and evaluating the models, leaving a total of 56784 events (exon-tissue pairs) from 7982 exons with at least one high-confidence measurement. See Additional file 1: Sections 1 and 2 of [4] for details on data processing.

Some of the additional analysis used tissue-averaged *Ψ* values, which are calculated from the full 16 × 10689 measurements. Analysis of mammalian conservation used phastCons basewise conservation scores [26] from 33 placental mammals; analysis of primate conservation used phastCons scores from 10 primates.

Deep neural network (DNN) models were trained using the Hebel Python/CUDA library [27]. This GPU-accelerated library performs backpropagation [28] via mini-batch stochastic gradient descent with Nesterov momentum [29] and L1 and L2 weight decay [30]. Hebel incorporates two recent breakthroughs in deep learning, dropout [31] and rectified linear units [32, 33], which have enabled DNNs to achieve state-of-the-art performance in a wide variety of problem domains, including speech recognition [34] and computer vision [35]. The architecture and training procedure of ConsNet (Additional file 1: Figure S1) and the other neural network models are described in Additional file 1: Section 4.

AUC, or area under the receiver-operating characteristic [36], denotes the ability of each model to discriminate between exons with *Ψ*< 1/3 from those with *Ψ*> 2/3; as shown in Fig. 3, these two groups collectively constitute 98.8 % of all exons. (The remaining 1.2 % of exons with intermediate *Ψ* were excluded from the AUC calculation since only a small deviation from the experimental value could cause these exons to be labelled as an incorrect prediction).

## Additional files

Additional file 1 Supplementary information. (PDF 183 kb)

Additional file 2 Upstream and downstream ISSs and ISEs. (CSV 1 kb)

Additional file 3 ISREs with high affinity for RBPs. (CSV 1 kb)

## Abbreviations

DNN: Deep neural network
ISE: Intronic splicing enhancer
ISRE: Intronic splicing regulatory element
ISS: Intronic splicing silencer
NMD: Nonsense-mediated decay
RBP: RNA binding protein
*Ψ*: Percent spliced-in
