# Supplementary material for: Does conservation account for splicing patterns?
Source: BMC Genomics. 2016 Oct 7;17:787. doi: 10.1186/s12864-016-3121-4 (PMC5055659; doi:10.1186/s12864-016-3121-4)
Supplement: Additional file 1 — Supplementary information. (PDF 183 kb) [file 12864_2016_3121_MOESM1_ESM.pdf]

Supplementary Material - Does conservation account for splicing patterns?

Differential regulation of Old$^+$ exons

There are two reasons Old$^+$ exons could have lower Ψ than Old$^-$: increased $\Delta\Psi$ across tissues, or lower Ψ in every tissue. To ascertain which effect has a greater contribution, we expanded our analysis to a dataset of Ψ values for 52 human and mouse cell types from Irimia et al. (2014) in order to capture as much regulatory diversity as possible. 80% of exons in this dataset which are Old$^+$ both upstream and downstream have $\Delta\Psi > 1/3$ between some pair of cell types, and this percentage could increase further as more cell types are considered (Fig. S1).

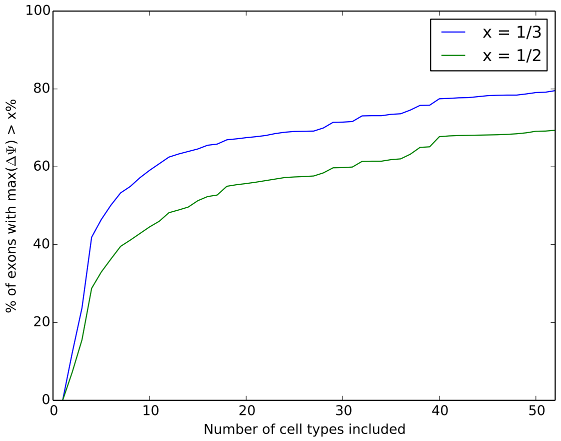

Figure S1: Percentage of Old$^+$ exons with $\Delta\Psi > x$ between any pair of cell types for various x, as increasing numbers of cell types are added to the analysis in random order, averaged over 100000 randomizations. Error bars are not shown since standard error is negligible. Because the percentages have not completely plateaued by 52 cell types, the true percentages could be somewhat larger when considering all possible cell types and conditions.

Tables of ISREs

The following four tables list ISE and ISS 6-mers. 6-mers with a high affinity for at least one RBP (see next section) are **bolded**. 6-mers identified as ISREs by Yeo et al. (2007) are *italicized*.

Table S1: Upstream ISEs ($n = 7$)

6-mer	q
CATTAC (GUAAUG)	0.0005
TTAATG (AAUUAC)	0.003
TTACGC (AAUGCG)	0.02
GAGCAA (CUCGUU)	0.03
**TTTGAA (AAACUU)**	0.04
AGCAGC (UCGUCG)	0.05
TTTTAA (AAAAUU)	0.05

Table S2: Upstream ISSs ($n = 35$)

6-mer	q
GGACTA (CCUGAU)	7e-07
GTAGCT (CAUCGA)	7e-07
**AGTAGC (UCAUCG)**	2e-05
**GAGTAG (CUCAUC)**	2e-05
GCTAAT (CGAUUA)	2e-05
TCAGCC (AGUCGG)	7e-05
TAGCTG (AUCGAC)	0.0001
**CGAGTA (GCUCAU)**	0.0003
TACAGG (AUGUCC)	0.0009
CTCAGC (GAGUCG)	0.002
AGCTGG (UCGACC)	0.003
GGGACT (CCCUGA)	0.003
CCTCAG (GGAGUC)	0.003
ACCACG (UGGUGC)	0.003
TGGGAC (ACCCUG)	0.003
**CCACCA (GGUGGU)**	0.004

GCCTCA (CGGAGU)	0.004
**CACCAC (GUGGUG)**	0.004
GACTAC (CUGAUG)	0.005
GGTTCA (CCAAGU)	0.005
CTGGGA (GACCCU)	0.005
GGCTAA (CCGAUU)	0.02
AGAGTT (UCUCAA)	0.02
AGCTAA (UCGAUU)	0.02
ACTACA (UGAUGU)	0.02
TGAGTA (ACUCAU)	0.02
**CCTCCC (GGAGGG)**	0.03
**GGTAAT (CCAUUA)**	0.03
CTACAG (GAUGUC)	0.03
CCACGC (GGUGCG)	0.03
**GTGTGC (CACACG)**	0.03
AGCCTC (UCGGAG)	0.04
CTCCCA (GAGGGU)	0.04
**TAAATC (AUUUAG)**	0.05
**CGGTAT (GCCAUA)**	0.05

Table S3: Downstream ISEs ($n = 16$)

**6-mer**	**q**
GATATT (CUAUAA)	0.003
TCTGAA (AGACUU)	0.005
CTGGCA (GACCGU)	0.01
CATCTA (GUAGAU)	0.01
TTAAGA (AAUUCU)	0.02
**AATAGG (UUAUCC)**	0.02
GGCTCT (CCGAGA)	0.02
AGTCCT (UCAGGA)	0.02
**CTTTTT (GAAAAA)**	0.03
**TGATAT (ACUAUA)**	0.03
CCAGTC (GGUCAG)	0.03
CTGAAG (GACUUC)	0.03
**AACGTA (UUGCAU)**	0.04
GCAGGA (CGUCCU)	0.04
ACTTTT (UGAAAA)	0.04
ATTTTA (UAAAAU)	0.04

Table S4: Downstream ISSs ($n = 85$)

**6-mer**	**q**
GCTAAT (CGAUUA)	7e-11
TGGTCT (ACCAGA)	6e-10
TAGAGA (AUCUCU)	6e-10
CTAATT (GAUUAA)	6e-10
AACTCC (UUGAGG)	6e-10
CTGGTC (GACCAG)	1e-09
GCTGGT (CGACCA)	5e-09
GTAGAG (CAUCUC)	1e-08
CATGTT (GUACAA)	3e-06
GTCTTG (CAGAAC)	5e-06
ATGTTG (UACAAC)	6e-06
AGAGAC (UCUCUG)	7e-06
ACTCCT (UGAGGA)	8e-06
GGCTAA (CCGAUU)	1e-05
AGGCTG (UCCGAC)	2e-05
GTGATC (CACUAG)	2e-05
TTGTAG (AACAUC)	2e-05
CAGCTA (GUCGAU)	2e-05
**CCATGT (GGUACA)**	2e-05
GAGACA (CUCUGU)	5e-05
TTTGTA (AAACAU)	0.0002
AGAGAT (UCUCUA)	0.0002
GGTTTC (CCAAAG)	0.0003
GTTGCC (CAACGG)	0.0004
**TGGCTA (ACCGAU)**	0.0006
CAGGCT (GUCCGA)	0.0006
**TAGTAG (AUCAUC)**	0.0008
GTTTCA (CAAAGU)	0.0009
TTGCCC (AACGGG)	0.001
CTCGAA (GAGCUU)	0.002
TCTTGA (AGAACU)	0.002
GGGTTT (CCCAAA)	0.003
**AGTAGA (UCAUCU)**	0.003

ACCATG (UGGUAC)	0.004
**TGTAGA (ACAUCU)**	0.004
GGTCTC (CCAGAG)	0.005
ATGGGG (UACCCC)	0.006
AGATGG (UCUACC)	0.006
TTCACC (AAGUGG)	0.007
GAACTC (CUUGAG)	0.007
CTTGAA (GAACUU)	0.007
TGACCT (ACUGGA)	0.007
GACGGG (CUGCCC)	0.007
**AAACTC (UUUGAG)**	0.007
TGTTGC (ACAACG)	0.007
GAGACG (CUCUGC)	0.007
AGATCA (UCUAGU)	0.007
GTATTT (CAUAAA)	0.007
GTCTCG (CAGAGC)	0.008
GCTCAA (CGAGUU)	0.01
GGCTGG (CCGACC)	0.01
TCAAAC (AGUUUG)	0.01
CACCAT (GUGGUA)	0.01
GGGGTT (CCCCAA)	0.01
CTACAT (GAUGUA)	0.01
CAAACT (GUUUGA)	0.01
**TTTTGT (AAAACA)**	0.01
TTTCAC (AAAGUG)	0.01
CCCAGC (GGGUCG)	0.02
GGTCTT (CCAGAA)	0.02
TCGCCA (AGCGGU)	0.02
ATTAGA (UAAUCU)	0.02
TTGTAT (AACAUA)	0.02
**TACTAA (AUGAUU)**	0.02
TCTCGA (AGAGCU)	0.02
TGGCCA (ACCGGU)	0.02
AGACAA (UCUGUU)	0.03
**ATACAA (UAUGUU)**	0.03
TCATTC (AGUAAG)	0.03
TCGAGA (AGCUCU)	0.03
AGCTAA (UCGAUU)	0.03
CAATAA (GUUAUU)	0.03
CAAGCG (GUUCGC)	0.03
GTTGGC (CAACCG)	0.04
ATTAGC (UAAUCG)	0.04
ACTCGT (UGAGCA)	0.04
TCGAAC (AGCUUG)	0.04
**CTCCTG (GAGGAC)**	0.04
CTCAAG (GAGUUC)	0.04
CAGCCT (GUCGGA)	0.04
CTTGAC (GAACUG)	0.04
CAAGCC (GUUCGG)	0.04
CATCAG (GUAGUC)	0.04
CCAGGC (GGUCCG)	0.05
AGACGA (UCUGCU)	0.05

RBPs with affinity for our ISREs

We catalogued 793 6-mers which contain the NOVA binding site YCAY (Dredge and Darnell 2003) or are subsequences of 7-mers found by Ray et al. (2013) to have a high affinity (E score > 0.45) for at least one of 207 RBPs. The following two tables list upstream and downstream ISREs which match these 793 6-mers, and the RBPs which have a high affinity for each one, with the affinity listed in brackets after each RBP. NOVA was not studied by Ray et al., so its affinities are not listed. ISEs are shown in green and ISSs in red.

Table S5: Upstream ($n = 12$)

**6-mer**	**RBPs and *E* scores**
TAAATC (AUUUAG)	Lm_0212 (0.475)
CACCAC (GUGGUG)	BRU-3 (0.475)<br>ARET (0.455)
CCTCCC (GGAGGG)	HRB98DE (0.469)<br>SF2 (0.464)<br>HNRNPH2 (0.458)
TTTGAA (AAACUU)	Tb_0219 (0.468)
GTGTGC (CACACG)	NCU08034 (0.466)<br>CPO (0.450)
CCACCA (GGUGGU)	BRU-3 (0.457)
AGTAGC (UCAUCG)	Tb_0216 (0.455)<br>NOVA
CGAGTA (GCUCAU)	NOVA
CGGTAT (GCCAUA)	NOVA
GAGTAG (CUCAUC)	NOVA
GGTAAT (CCAUUA)	NOVA
TGAGTA (ACUCAU)	NOVA

Table S6: Downstream ($n = 14$)

**6-mer**	**RBPs and *E* scores**
AACGTA (UUGCAU)	RBFOX1 (0.488)<br>FOX-1 (0.483)<br>ASD-1 (0.481)<br>A2BP1 (0.473)<br>MAL13P1.35 (0.450)
TGTAGA (ACAUCU)	Lm_0212 (0.467)
AAACTC (UUUGAG)	Ng_0261 (0.466)
ATACAA (UAUGUU)	EXC-7 (0.464)
CTTTTT (GAAAAA)	PABPC4 (0.463)<br>PABP (0.460)<br>Tv_0257 (0.455)
TTTTGT (AAAACA)	PABPC4 (0.461)
CCATGT (GGUACA)	MAL13P1.35 (0.458)
AATAGG (UUAUCC)	Tb_0219 (0.458)
CTCCTG (GAGGAC)	SF2 (0.456)
TACTAA (AUGAUU)	HuR (0.456)
TGGCTA (ACCGAU)	PF10_0214 (0.455)
TGATAT (ACUAUA)	Tb_0219 (0.454)
AGTAGA (UCAUCU)	NOVA
TAGTAG (AUCAUC)	NOVA

Architecture and training procedure of neural network models

Fig. S2 shows the architecture of ConsNet, the neural network model that achieves state-of-the-art performance at absolute Ψ prediction. ConsNet predicts Ψ from sequence and conservation data via the following steps:

1. The raw genetic sequence, represented as a 4-by-sequence-length binary matrix, is **convolved** with n filters of width w, analogous to position-weight matrices (Stormo et al. 1982), to give a 1-by-sequence-length 'feature map' for each filter indicating the similarity of that filter to each point in the sequence. While the filters do not necessarily have a direct biological interpretation, they can represent, for instance, consensus motifs for splice sites or *cis*-acting RBPs.

2. Each of the n feature maps is shifted upwards by a **bias** that depends on the filter, then **rectified** to set negative values (i.e. regions of the sequence that do not correspond well to the filter) to zero. The purpose of this step is to apply a non-linearity to the feature map, which increases the modelling capacity of the network.

3. Each feature map is **weighted by conservation**, specifically the phastCons score at each base, which intuitively has the effect of focusing the network's attention on the most conserved regions of the sequence. However, since conservation turns out to be much more useful than the sequence itself, it might be better to think of this step as weighting conservation by the sequence, rather than the other way around.

4. Each feature map is **max pooled** over a certain pooling width: only the largest value of the feature map in each region of the sequence is retained, and the other values are discarded.

5. Features from the pooled feature maps of every filter pass through several **hidden layers**, where each layer is a non-linear function of the previous layer. Here, important features of the max-pooled feature map are recombined and distilled into composite features.

6. The composite features of the last hidden layer are combined to predict each tissue's Ψ, using **multi-task learning**: each tissue takes a different linear combination of the features, then applies the sigmoid function $\sigma(x) = \frac{1}{1+e^{-x}}$ so that the result is between 0 and 1.

The convnet of Fig. 1(a) has the same architecture as ConsNet except that the conservation weighting step is omitted. Convnets, or convolutional neural networks, are a standard type of neural network architecture traditionally used for image processing (Fukushima 1988; LeCun et al. 1998). All other networks used a standard feed-forward architecture, with input data rescaled to have zero mean and unit variance to improve classification performance.

Informally, a model is trained by repeatedly showing it the sequence and conservation data for randomly chosen exons and asking it to predict their splicing levels in each tissue. If a prediction differs from the experimental value, every parameter in the model – the filters and their biases, as well as the components of the non-linear functions for combining the feature maps into a prediction – is changed simultaneously in the direction (increase or decrease) that would reduce the error in the prediction, in proportion to the effect that this change would have in reducing the error.

The 7982 exons where at least one tissue had a high-confidence Ψ measurement were randomly divided into 3/4 training data, 1/8 validation data and 1/8 test data. Model settings, or 'hyperparameters', were selected via random search (Bergstra and Bengio 2012) to optimize performance on the validation data; AUCs are reported for predictions on the test set concatenated over all tissues.

Hyperparameters

The following hyperparameters were searched over for each of the experiments in Fig. 1:

- *Num hidden layers*: the number of hidden layers (search range: 0 to 4, or 0 to 3 for (h) and (i))
- *Num hidden units*: the number of hidden units in each layer (50, 100, 200, 500 or 1000, with the restriction that each layer must not have more units than the previous)
- *Learning rate (LR)*: the initial learning rate
- *LR decay*: the value the learning rate is multiplied by after each epoch, so that it decays exponentially (0.97 to 1)
- *LR multiplier - output*: the multiple of the learning rate used for the output layer (0.2 to 5, log scale)
- *LR multiplier - hidden*: the multiple of the learning rate used for the hidden layers (0.2 to 5, log scale)
- *Weight scale - hidden*: the scale of the random weight initializations of the hidden layers, which are chosen uniformly between this number and its negative (0.001 to 0.1, log scale)

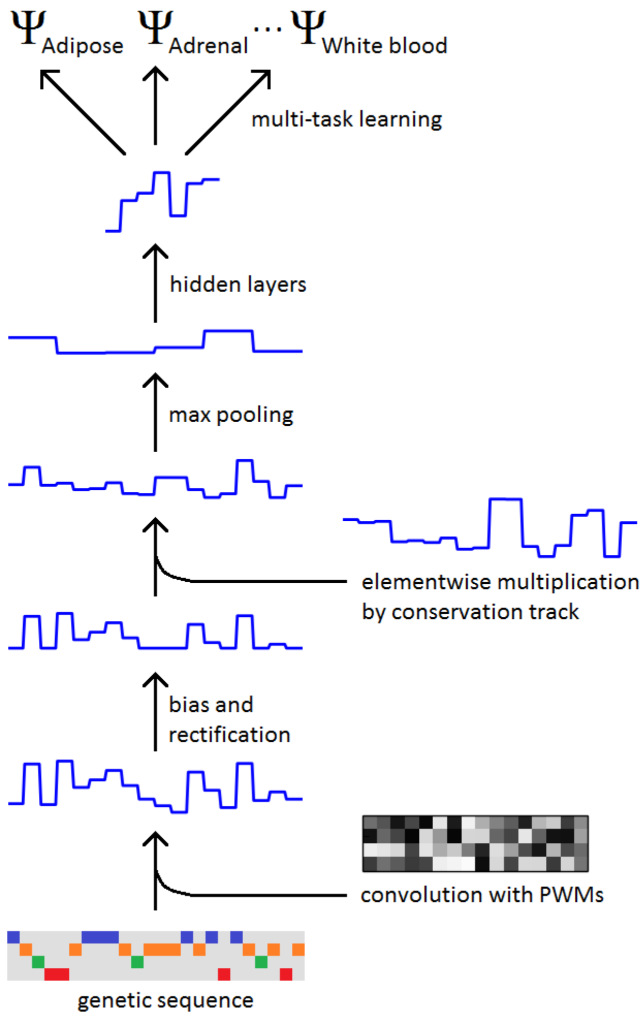

Figure S2: Architecture of ConsNet, a deep neural network model that achieves state-of-the-art performance at predicting alternative splicing.

- *L1 weight penalty*: the error penalty for the magnitude of each weight (1e-10 to 1e-3, log scale)
- *L2 weight penalty*: the error penalty for the squared magnitude of each weight (1e-10 to 1e-3, log scale)
- *Dropout probability*: the chance of removing each hidden unit for each minibatch during training (0 to 0.5 in steps of 0.1)
- *Momentum*: starts at *Initial momentum* (0.05 to 1, 1 - log scale), increases linearly to *Plateau momentum* (0.001 to 1, 1 - log scale) over *Increase time* epochs (0 to 40) to give the training procedure time to find a direction of consistent gradient, stays constant until *Plateau end* epochs (40 to 80), then decreases linearly to *Final momentum* (0.05 to 1, 1 - log scale) over *Decrease time* (10 to 60) epochs to avoid overshooting the optimum. Note that the momentum is not mandated to first increase and then decrease; rather, the search usually selects trials with this trajectory. For most trials, early stopping occurs before *Decrease time*, so subsequent momentum hyperparameters are not listed.
- *Early stopping epoch*: the epoch at which the maximum validation error is obtained (0 to 100)

The weight scale of the output layer was not searched over, and was instead selected according to the *normalized initialization* formula from Glorot and Bengio (2010). A minibatch size of 100 was used for all trials.

Several additional hyperparameters were searched over for the convnet and ConsNet:

- *Pooling width*: the width of the pooling regions used in max pooling (3, 6, 12 or 24). (a) and (b) also used features from an intermediate pooling step (48, 96, 192), although this did not significantly affect performance and was removed for (c) and (d).
- *Num pooling regions*: the number of pooling regions nearest the splice site used as features (4, 8, 12 or 16; for the intermediate pooling step, 2, 4, 6 or 8)
- *Filter width*: the width of the convolutional filters (6 to 32)
- *Num filters*: the number of filters (24 to 128)
- *Weight scale - conv*: the scale of the random weight initializations of the convolutional layer (0.001 to 0.1, log scale)
- *LR multiplier - conv*: the multiple of the learning rate used for the convolutional layer (0.2 to 5, log scale)

The optimal hyperparameters found for each experiment are shown in Table S7.

	(a) Convnet	(b) ConsNet	(c) ConsNet exons	(d) ConsNet introns	(f) Cons	(g) Junc/Avg	(h) Junc	(i) Avg	(j) (e)+(g)
Num hidden layers	1	2	1	1	2	2	1	1	1
Num hidden units	100	50<br>50	1000	500	1000<br>50	50<br>50	500	200	500
Weight scale - hidden	0.017	0.063<br>0.045	0.029	0.034	0.002<br>0.052	0.017<br>0.001	0.016	0.002	0.002
L1 weight penalty	1.9e-7	4.4e-6	6.7e-10	5.9e-10	1.3e-10	1.6e-7	9.5e-10	6.9e-8	1.6e-4
L2 weight penalty	6.0e-4	7.3e-5	2.6e-6	1.8e-9	6.2e-10	6.8e-5	1.0e-10	7.8e-5	4.1e-9
LR multiplier - output	0.220	2.070	2.770	0.238	2.827	0.204	0.234	0.213	2.216
LR multiplier - hidden	2.103	0.215	0.269	1.932	0.784	2.548	0.998	0.922	0.251
Dropout	True	True	True	False	True	False	False	False	True
Learning rate (LR)	0.049	0.074	0.091	0.059	0.010	0.309	0.022	0.316	0.091
LR decay	0.992	0.970	0.975	0.998	0.973	0.988	0.988	0.975	0.977
Initial momentum	0.931	0.528	0.545	0.925	0.789	0.925	0.875	0.949	0.943
Plateau momentum	0.995	0.983	0.976	0.714	0.989	0.987	0.999	0.979	0.529
Final momentum	–	0.376	–	–	–	–	0.455	–	–
Increase time	26	15	29	19	22	3	9	2	5
Plateau end	–	51	–	–	–	–	47	–	–
Decrease time	–	41	–	–	–	–	52	–	–
Pooling width	48<br>6	192<br>12	24	12	–	–	–	–	–
Num pooling regions	8<br>16	4<br>12	4	8	–	–	–	–	–
Filter width	10	12	13	6	–	–	–	–	–
Num filters	79	58	128	53	–	–	–	–	–
Weight scale - conv	0.064	0.001	0.020	0.073	–	–	–	–	–
LR multiplier - conv	1.940	1.016	2.524	0.714	–	–	–	–	–
Early stopping epoch	60	65	40	60	45	35	50	45	5

Table S7: Optimal hyperparameters for each of the experiments in Fig. 1, as selected by random search. 1(e), the previous state of the art, is not shown.

References

Irimia, M, Weatheritt, RJ, Ellis, JD, Parikshak, NN, Gonatopoulos-Pournatzis, T, Babor, M, Quesnel-Vallières, M, Tapial, J, Raj, B, OHanlon, D, et al. A highly conserved program of neuronal microexons is misregulated in autistic brains. 2014. *Cell* **159:** 1511–1523.

Yeo, GW, Van Nostrand, EL, and Liang, TY. Discovery and analysis of evolutionarily conserved intronic splicing regulatory elements. 2007. *PLoS Genetics* **3:** e85.

Dredge, BK and Darnell, RB. Nova regulates GABAA receptor $\gamma 2$ alternative splicing via a distal downstream UCAU-rich intronic splicing enhancer. 2003. *Molecular and cellular biology* **23:** 4687–4700.

Ray, D, Kazan, H, Cook, KB, Weirauch, MT, Najafabadi, HS, Li, X, Gueroussov, S, Albu, M, Zheng, H, Yang, A, et al. A compendium of RNA-binding motifs for decoding gene regulation. 2013. *Nature* **499:** 172–177.

Stormo, GD, Schneider, TD, Gold, L, and Ehrenfeucht, A. Use of the 'Perceptron' algorithm to distinguish translational initiation sites in E. coli. 1982. *Nucleic Acids Research* **10:** 2997–3011.

Fukushima, K. Neocognitron: A hierarchical neural network capable of visual pattern recognition. 1988. *Neural Networks* **1:** 119–130.

LeCun, Y, Bottou, L, Bengio, Y, and Haffner, P. Gradient-based learning applied to document recognition. 1998. *Proceedings of the IEEE* **86:** 2278–2324.

Bergstra, J and Bengio, Y. Random search for hyper-parameter optimization. 2012. *The Journal of Machine Learning Research* **13:** 281–305.

Glorot, X and Bengio, Y. Understanding the difficulty of training deep feedforward neural networks. 2010. *AISTATS***:** 249–256.
